# Aphid species specializing on milkweed harbor taxonomically similar bacterial communities that differ in richness and relative abundance of core symbionts

**DOI:** 10.1038/s41598-022-25554-y

**Published:** 2022-12-07

**Authors:** Laramy Enders, Thorsten Hansen, Kirsten Brichler, John Couture, Elizabeth French

**Affiliations:** 1 Department of Entomology, Purdue University, West Lafayette, IN USA; 2 Southwest Minnesota State University, Marshall, MN USA

**Keywords:** Microbial communities, Microbial ecology, Microbiome, Symbiosis, Microbial ecology

## Abstract

Host plant range is arguably one of the most important factors shaping microbial communities associated with insect herbivores. However, it is unclear whether host plant specialization limits microbial community diversity or to what extent herbivores sharing a common host plant evolve similar microbiomes. To investigate whether variation in host plant range influences the assembly of core herbivore symbiont populations we compared bacterial diversity across three milkweed aphid species *(Aphis* *nerii, Aphis asclepiadis, Myzocallis asclepiadis*) feeding on a common host plant (*Asclepias syriaca*) using 16S rRNA metabarcoding. Overall, although there was significant overlap in taxa detected across all three aphid species (i.e. similar composition), some structural differences were identified within communities. Each aphid species harbored bacterial communities that varied in terms of richness and relative abundance of key symbionts. However, bacterial community diversity did not vary with degree of aphid host plant specialization. Interestingly, the narrow specialist *A.* *asclepiadis* harbored significantly higher relative abundances of the facultative symbiont *Arsenophonus* compared to the other two aphid species. Although many low abundance microbes were shared across all milkweed aphids, key differences in symbiotic partnerships were observed that could influence host physiology or additional ecological variation in traits that are microbially-mediated. Overall, this study suggests overlap in host plant range can select for taxonomically similar microbiomes across herbivore species, but variation in core aphid symbionts within these communities may still occur.

## Introduction

Establishing partnerships with microbes is hypothesized to enhance the adaptive potential of the host organism^1–4^. In fact, both plants and insects harbor diverse microbial communities that provide unique advantages, including protection from stress and novel mechanisms for nutrient acquisition^4–7^. However, only recently have microbes come to be viewed as mediators of plant–insect interactions. From an insect perspective, microbes can be critical in facilitating or restricting the use of host plants by aiding in digestion of plant material and detoxification of anti-herbivore chemical defenses^3,5,8^. Soil and root-associated microbes also play essential roles in plant growth and defense against both above and belowground insect attackers^6,7,9,10^. Although microbes unquestionably impact plant–insect interactions, the ecological factors and selective forces shaping plant and insect-associated microbial communities remain poorly understood.

One factor thought to play a pivotal role in shaping insect herbivore microbiomes is host plant range or diet breadth, which can vary from a single species to hundreds of plant species^11–16^. Several plant associated factors, including nutritional quality and defensive chemistry, are known to influence insect microbial community dynamics by selecting for taxa that facilitate enhanced colonization and survival^17^. For example, associations with heritable bacterial symbionts have long been recognized as enabling phloem feeding insects to exploit an otherwise poor nutritional source^17,18^. Microbes are also hypothesized to contribute to variation in the capacity of insect herbivores to consume chemically defended plants^8^. Finally, feeding on different host plants can influence the composition of microbial communities associated with insect herbivores^11,14,19,20^. However, aside from a handful of well-characterized nutritional symbionts it is unclear to what extent insect microbial communities either directly or indirectly contribute to host plant adaptation and diet breadth. Furthermore, the mechanisms contributing to variation in herbivore microbiomes and ecological implications for plant–insect coevolution are not well understood^14^.

Insect microbiomes consist of varying combinations of heritable symbionts and flexible pools of environmentally acquired microbes^21–23^, but how host plant range shapes the taxonomic and functional diversity of these communities is relatively unexplored^5,24–26^. Variation in host plant range can influence the assembly of microbial communities and dynamics of symbiotic partnerships via several hypothesized mechanisms. For example, microbial communities of specialist herbivores are hypothesized to be less diverse relative to generalist species that possibly require a larger microbial repertoire to successfully colonize highly variable hosts (e.g. adaptive advantage to harboring multiple heritable symbionts)^5,24^. Generalists may also be exposed to and therefore acquire greater environmental microbial diversity from feeding on a broader host plant range. Alternatively, overlapping host plant range across herbivore species could result in selection for common microbes needed for successful colonization due to similarities in nutritional ecology or defensive chemistry of shared host plants (i.e. purifying selection reduces differences in symbiont communities). Currently, it is unclear whether broader patterns associated with host plant use exist, such as a gradient in microbiome diversity from specialist to generalist herbivores or shifts in abundance of key heritable symbionts.

Heritable bacterial symbionts are hypothesized to expand the host plant range of sap-feeding insects, particularly aphids^26–28^. Signatures of host plant specificity have been detected in aphid microbiome composition^20,29–32^. Even within a single aphid species ecologically divergent biotypes specialized on different host plants can harbor distinct microbiomes that differ in taxonomic composition and frequency of symbionts^25,33,34^. In contrast, recent work suggests aphid phylogeny (i.e. species relatedness) and geographic isolation of populations are dominant factors predicting differences in bacterial communities, while host plant range may be less important overall^21–23^. However, aphid studies have primarily focused on the role microbial symbionts play in host plant use by highly polyphagous species, while much less is known about monophagous or oligophagous species. As a result, the relative importance of ecological factors such as host plant range and geographic range in governing aphid symbiont community assemblages remains unclear.

The current study aims to address the following question: How does variation in host plant range shape the assembly of aphid symbiont communities? Aphids colonizing milkweed species in the subfamily *Asclepiadaceae* exhibit variation in host plant specialization^35^ and thus represent an excellent system in which to investigate the role diet breadth plays in shaping aphid microbial community composition. *Aphis* *nerii* is considered a broad specialist capable of feeding on over 50 plant species, including oleander, milkweed, and periwinkle. *Aphis* *asclepiadis* is a narrow specialist, feeding on less than 10 A*sclepias* spp., and *Myzocalis asclepiadis* is monophagous, feeding only on the common milkweed A*sclepias syriaca*^35,36^. Currently, few populations have been screened for symbionts and thus the bacterial community of milkweed aphids remains largely unexplored^37^. One possibility is that milkweed aphid species have few microbial symbionts besides the universal obligate symbiont *Buchnera* due to the toxicity of milkweed defensive chemicals (e.g. cardenolides), which when ingested could make the internal aphid environment inhospitable for sustained microbial growth (e.g. via direct anti-microbial effects). However, it is generally unclear how ecological factors (e.g. host plant range, geographic location) shape both the heritable and environmentally acquired microbiota of milkweed aphids. We therefore characterized the diversity and composition of bacterial communities using targeted amplicon sequencing of field collected aphids found naturally colonizing common milkweed (*A.* *syriaca*). Specifically, we tested two alternative hypotheses regarding the role host plant range plays in shaping aphid symbiont community assembly. First, symbiont diversity (i.e. bacterial species richness) is hypothesized to increase with expansion of host plant range or increased diet breadth (*A.nerii* > *A.* *asclepiadis* > *M.* *asclepiadis*). Alternatively, overlap in host plant range is hypothesized to reduce differences in symbiont communities across aphid species (e.g. via purifying selection from exposure to similar plant defensive chemicals). Overall, we find that milkweed aphids collected from a common host plant tend to have highly similar bacterial microbiomes when considering the broader pool of taxa detected across populations, but differences in relative abundances and strain diversity were found in core heritable symbionts.

## Methods

### Aphid field collection & sample preparation

In this study we focus on three aphid species (*A.nerii*, *A.* *asclepiadis, M.* *asclepiadis*) that colonize plants of the family Apocynaceae with varying degrees of host specialization^35,36^. The broad specialist species *A.nerii* is considered an obligate parthenogen (only reproduces asexually in the wild) and in its introduced range in the United States (U.S.) it colonizes species of Apocynoideae (oleander) and Asclepiadaceae (milkweed)^38,39^. The wide host plant distributions for *A.* *nerii* overlap and extend into northern and midwestern regions of the U.S.^40,41^. Therefore populations of *A.nerii* can potentially feed on multiple host plant species throughout the summer in the midwestern and eastern U.S. However, *A. nerii* cannot tolerate freezing temperatures and is unable to support year round populations except in the southernmost regions of the U.S.^38,39^. The narrow specialist *A.* *asclepiadis* is also broadly distributed across the eastern and central U.S., but it is unknown if this species is an obligate or cyclical parthenogen or whether it can overwinter in the egg stage in northern regions^39^. Even less is known about the life history of the monophagous species *M.* *asclepiadis*, which may be cyclically parthenogenic and overwinter in the egg stage. Interestingly, *M.* *asclepiadis* is less gregarious compared to the other two species and highly mobile due to the asexual production of only winged adults during the summer^36,42^.

To address our central question and test hypotheses regarding differences in aphid microbiomes associated with degree of host plant specialization, we sampled aphids from a single common host plant across different locations. By sampling from a single host plant type we focus on detecting differences in heritable symbionts associated with variation in aphid diet breadth, and to a lesser extent environmentally acquired microbes associated with different locations or individual host plants. Our sampling design avoids confounding effects associated with variation in microbiota that could be acquired from feeding on different host plant species, and instead focuses on detection of variation in the heritable fraction of the aphid microbiome (e.g. broader host plant ranges may select for greater variation in heritable facultative symbiont populations). Further, *M.* *asclepiadis* is monophogus and it is not possible in this system to sample all three aphid species from multiple shared host plant species, an experimental design that may be feasible in other systems to further tease apart host plant-aphid-microbiome interactions.

Aphids were collected in July–Aug 2017 from a single host plant type, the common milkweed (*A.* *syriaca)*, across 14 locations generally surrounding the Purdue campus (Supplementary Table S1) in West Lafayette, IN. *Ascelpiadis syriaca* is among the most abundant milkweed species in the midwestern United States^43^. Locations were chosen based on the presence of large patches of common milkweed in open grassy fields away from roadsides. Distance between locations ranged from a minimum of 1 km to a maximum of 141 km (Supplementary Table S1). To avoid sampling heavily from single aphid clonal families (e.g. offspring from a single alate aphid colonizing a plant) that could limit the ability to capture natural variation in microbial communities, aphids were collected from 3 to 5 separate plants (< 20 m apart) within a single location (e.g. from distinct milkweed patches when possible) and pooled together into a single sample for downstream microbiome analysis. In some cases, 2 aphid species were found co-colonizing a single plant and thus single species versus mixed species samples were designated as such during data collection (Supplementary Table S1). Individual adult aphids were removed from milkweed leaves by hand using a paint brush, identified and separated by species using standard identification guides [^35^, http://www.aphidsonworldsplants.info/], and then placed in Eppendorf storage tubes containing 95% ethanol. Only adult aphids were included because they have distinct morphological characteristics that allow for straightforward species identification (e.g. body coloration; *M.* *acelepiadas* has distinctive red–orange blotches and black markings), thus preventing mixed species samples and ensuring pools of aphids used for downstream analysis were separate species. *A.nerii* and *A.* *asclepiadis* reproduce parthenogenetically during the summer and can produce winged individuals under crowded conditions^39^. Therefore, only wingless apterous adults were collected from *A. nerii* and *A.* *asclepiadis*, which are relatively sessile and typically remain on a single plant from birth to death unless disturbed. In contrast, *M.* *asclepiadis* only produce alates or winged individuals in the summer, which can move between individual plants of their only host *A.* *syriaca*^36^. Overall, individuals used in this study are unlikely to have feed on multiple host plant species prior to field collection. All individuals collected from each separate plant at each location were stored in 95% ethanol at −20C until further processing. The total number of individuals collected per species at a single location ranged from 10 individuals to > 50 individuals.

For bacterial community profiling, groups of 5 individual aphids per species were selected from the total pooled sample per location for DNA extraction and sequencing. Total DNA was extracted from groups of 5 whole aphids using the Qiagen DNeasy kit following standard protocols. Aphids were surface sterilized with 95% ethanol (during storage) and washed with ultra pure water prior to extraction. While bacterial DNA recovered is expected to be primarily from internal communities, it is possible not all external or cuticular bacterial DNA was eliminated and is expected to contribute to overall diversity estimates^44^. Total DNA concentration was measured using a Nanodrop spectrophotometer for all samples. In total 82 pooled aphid samples were processed and sequenced for microbiome analysis (*A.* *nerii* n = 46, *A.* *asclepiadis* n = 30, *M.* *asclepiadis* n = 6; Supplemental Table S1). Targeted amplicon sequencing was used to characterize the bacterial communities associated with the three milkweed aphid species (*A.* *nerii, A. asclepiadis, M. asclepiadis*) across the 19 locations sampled. Library preparation and sequencing was performed at the University of Minnesota Genomics Core Facility on an Illumina MiSeq instrument (V3 cluster chemistry, paired end 250 bp sequencing) following optimized methods that target the V4 region of the 16 s rDNA gene^45^. Primers used were standard V4 region primers 515F-GTGCCAGCMGCCGCGGTAA and 806R-GGACTACHVGGGTWTCTAAT^46^. All sequence data is available on NCBI SRA database under project number PRJNA635683.

### Characterization & analysis of aphid bacterial communities

Sample demultiplexing was done by the University of Minnesota Genomics Center with Illumina software. Trimmomatic [v. 0.36;^47^] and Cutadapt [v 1.13;^48^] were used to remove adapters and primer sequences and low quality reads. All subsequent processing was performed in R (v 3.6.3) and Bioconductor (v 3.10). Trimmed reads were processed through the dada2 [v 1.14.1;^49^] pipeline by filtering and trimming based on read quality, inferring error rates, merging paired end reads, removing chimeras, and assigning taxonomy with the Silva reference database v. 132. Removal of very low abundance reads was done using a cut-off of fewer than 10 reads in 5% of the samples. Next, eukaryotic and mitochondrial sequences were removed. Lastly, individual samples with fewer than 2500 reads were removed and a final sample-level filtering step (i.e. *Buchnera* ASVs with < 1% reads within a sample were removed from individual samples) was applied to the remaining 64 samples to account for potential read contamination or “cross-talk” among samples that can occur in metabarcoding studies of microbial communities that are dominated by few high abundance symbionts (e.g. *Buchnera*–^50^). We applied the more conservative exclusion cut-off to *Buchnera* ASVs only in order to avoid loss of rare community members and minimize false positives resulting from mis-binning *Buchnera* sequences. A summary of raw sequencing results and processing steps can be found in Supplementary Table S2.

Following sequence processing, all downstream analyses and data visualization was run in R (v 3.6.3). All code for statistical analyses and generation of figures, including information on R packages used, can be found in the Purdue University Github (https://github.itap.purdue.edu/LaramyEndersGroup/Milkweed-Aphid-Microbiome). Specifically, we compared standard alpha and beta diversity metrics using the phyloseq [v 1.30.0;^51^] and vegan [v 2.5–6;^52^] packages in R to determine the extent to which microbiomes varied in taxonomic composition and structure across aphid species and sampling locations. Initial data assessment (i.e. Q-Q plot of residuals, Shapiro–Wilk Normality test and Levene test for homogeneity of variance) indicated our alpha diversity data did not fit the assumptions of a linear model. To compare species richness and evenness we therefore used the non-parametric Kruskal–Wallis test to test the effect of aphid species on diversity metrics, followed by the Wilcoxon Rank Sum test with Holm correction for multiple testing to identify significant differences among aphid species. Differences in the structure of bacterial communities across aphid species was assessed through PERMANOVA analysis of beta diversity (Unifrac, weighted Unifrac, Bray–Curtis) and visualized using Non-Metric Multidimensional Scaling (NMDS). Homogeneity of dispersion across aphid species’ samples was also tested using PERMDISP. To identify differentially abundant bacterial sequences across aphid species we applied generalized linear mixed models to normalized read counts using DeSeq2 [v 1.26.0;^53^] and made pair-wise comparisons between each aphid species. Normalized read counts account for variation in library size and were used to estimate relative abundance of each ASV. Finally, we compared strain level genetic differences in *Buchnera* and *Arsenophonus* ASVs using a phylogenetic approach by aligning sequences with the DECIPHER package [v 2.14.0;^54^] and building Maximum-likelihood trees with the optim.pml(model = “GTR”, rearrangement = “stochastic”) function in the phangorn package in R [v 2.5.5;^55^].

## Results

**Figure 1:**
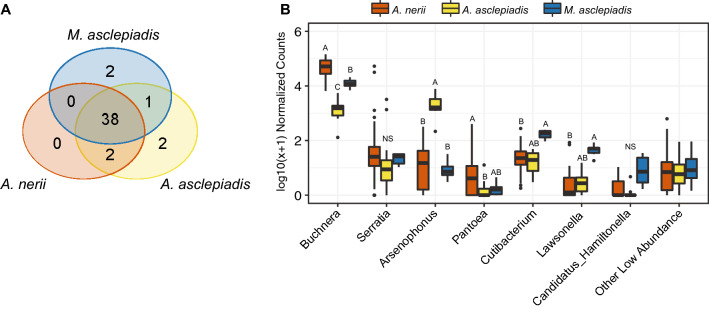
(**A**) Venn Diagram showing the extent to which amplicon sequence variants (ASVs) are shared and which are unique to each milkweed aphid species from samples collected across all locations. (**B**) log10(x + 1) normalized counts of bacterial taxa found across the three milkweed aphid species. If multiple strains were present they were grouped together under one genus. Very low abundant ASVs (below 1% relative abundance) were grouped together into the “Other” category, except for known aphid symbionts (i.e. *Hamiltonella*) and significantly differentially abundant taxa (see Supplementary Tables S3 & S4; Supplementary Fig. S2 for details). Differing letters indicate significantly differential abundance across species by DESeq2, with an adjusted *p* = 0.05. NS = not significant. Figure panels generated in R and joined using Adobe Illustrator (v.23.0.1).

Overall, most bacterial taxa identified through sequencing were found to occur in all three milkweed aphid species (Fig. 1a). In total 45 amplified sequence variants (ASVs) were identified (Supplementary Table S3), 38 of which occurred in each species when all sampling locations were considered. Bacterial communities in general were dominated by the primary aphid symbiont *Buchnera*, which was in high relative abundance compared to the remaining taxa (Fig. 1b). Among the non-*Buchnera* ASVs identified several well-known aphid facultative symbionts were found, including *Arsenophonus, Serratia,* and *Hamiltonella,* which occurred in all milkweed aphid species to varying degrees (Fig. 1b). In a few samples *Regiella, Ricketsiella,* and *Wolbachia* were identified in very low abundance (< 2 reads in 10% of samples) and did not make the cutoff to be included in analysis (Supplementary Fig. S1). Much of the remaining low-abundance taxa identified are likely environmentally acquired from the plant host or surrounding soil (e.g. *Pantoea*, *P*s*eudomonas,* *Streptococcus*). The prevalence or proportion of samples that tested positive for each ASV also tended to vary across aphid species (Supplementary Table S3). For example, *Buchnera* was found in all samples, but *Hamiltonella* ranged from 13% (2/15) prevalence in *A.* *asclepiadis* samples to 100% (6/6) in *M.* *asclepiadis* and 30% (13/43) in *A.* *nerii* samples. *Serratia* was also highly prevalent across all three species, ranging from 83% in *A.* *nerii* to 100% in *M.* *asclepiadis* samples*.* Interestingly, all three identified strains of *Lactobacillus* were found in 100% of *M.* *asclepiadis* samples but were only present in 50% or less of samples of the other two species. Co-occurring strains of *Buchnera* and *Arsenophonus* were also found to varying degrees in all three species, although it should be noted that we tested groups of aphids and it remains unknown whether individual aphids harbor different strains (i.e. multiple infections).

**Figure 2:**
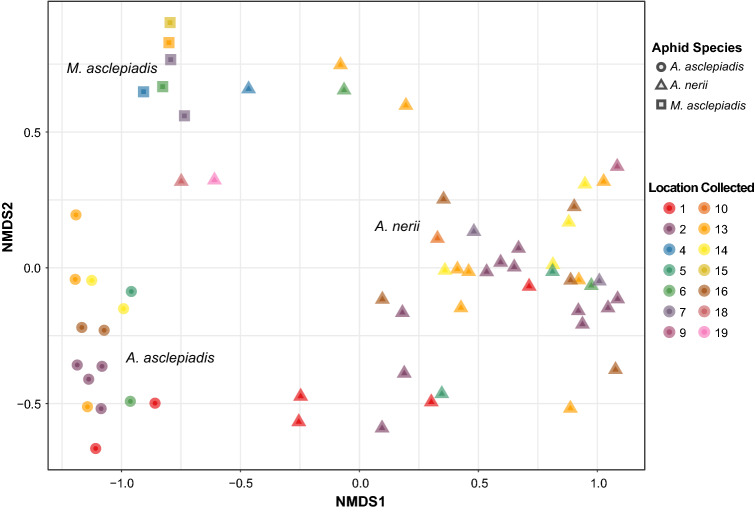
NMDS of Bray–Curtis dissimilarity showing how bacterial community structure varies across milkweed aphid species and location.

**Figure 3:**
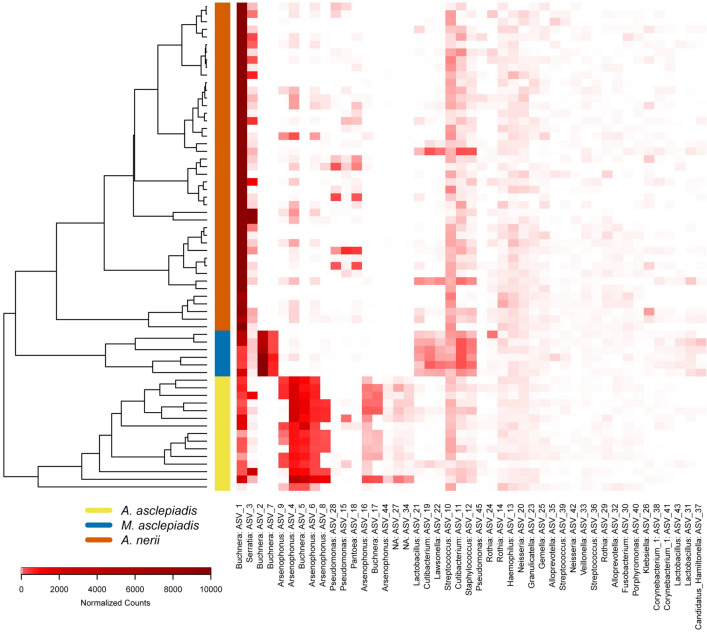
Hierarchical clustering of all samples based on DESeq2 normalized read counts of each bacterial taxa.

**Figure 4:**
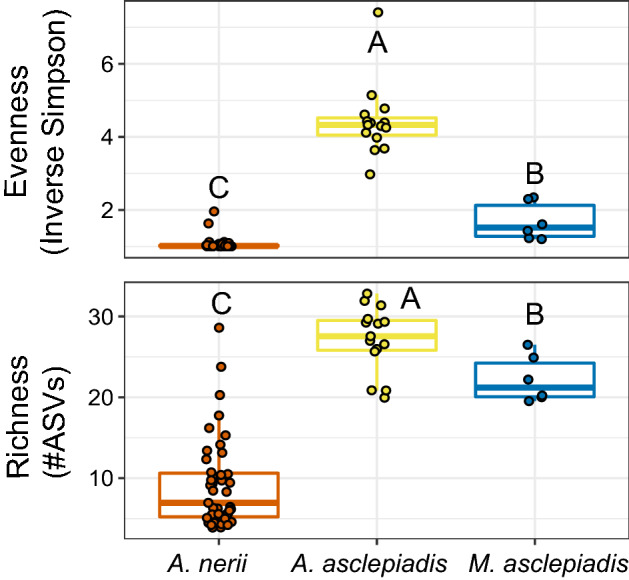
Species richness and evenness within aphid bacterial communities were compared across aphid species. Differing letters indicate significant differences at *p* < 0.05 by Wilcoxon Rank Sum Test (Supplementary Table S5).

Although most ASVs were shared at a global level when the entire pool of microbes was considered across locations (Fig. 1a), several key differences indicate that each milkweed aphid species harbors distinct bacterial communities that vary in structure and abundance of taxa (Figs. 1b, 2, Supplementary Table S4). NMDS of Bray–Curtis dissimilarity between samples (Fig. 2) indicates that bacterial community structure is unique to each aphid species (*p* < 0.001, Supplementary Table S5). Similar results were found for all beta diversity measures tested, including PERMDISP tests for homogeneity of dispersion across aphid species (Supplementary Table S5). Hierarchical clustering of samples by differences in community structure and relative abundance of each taxa further shows that each aphid species forms a unique group (Fig. 3). In addition to differences in overall community structure there was significant variation in the bacterial species richness and evenness of communities found across the three aphid species (Fig. 4, Supplementary Table S5). Interestingly, the broad specialist *A.* *nerii* had the lowest bacterial species richness and the more narrow specialist *A.* *asclepiadis* had the highest among the three species. Overall, results did not show a gradient in bacterial diversity (i.e. species richness) associated with diet breadth or degree of host-plant specialization across these three milkweed aphid species.

**Figure 5:**
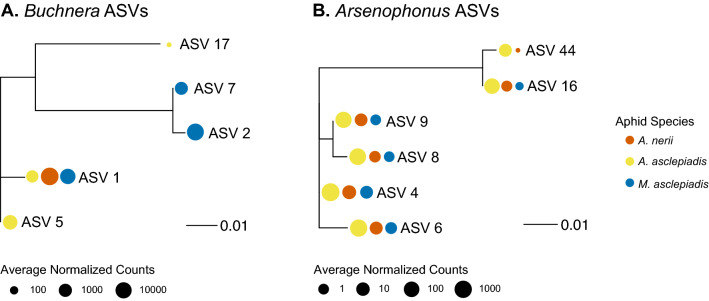
Genetic variation and differences in relative abundance of *Buchnera* and *Arsenophonus* strains (ASVs) identified in the three milkweed aphid species. All *Buchnera* and *Arsenophonus* ASVs were differentially abundant (adjusted *p* < 0.05) in at least one species comparison by DESeq2 (Supplementary Table S6). Figure panels generated in R and joined using Adobe Illustrator (v.23.0.1).

To further identify factors contributing to differences between aphid microbiomes we compared the relative abundance of individual taxa and variation in bacterial strain diversity (ASVs) (Supplementary Table S6; Figs. 3, 5). Differences in community structure were primarily driven by (1) variation in the composition of facultative symbionts and other low-abundance (non-*Buchnera*) bacteria (Figs. 1b, 3) and (2) the presence of *Buchnera* and *Arsenophonus* strains unique to each aphid species (Fig. 5). Overall, *A.* *nerii* bacterial communities were dominated by *Buchnera* with few other symbionts in low abundance, which differs from the other two aphid species. Unique to *A.* *asclepiadis* was the overall higher *Arsenophonus* abundances, but lower *Buchnera* abundances compared to the other two species (Fig. 1b). Each aphid species typically harbored a single dominant *Buchnera* strain in highest relative abundance, with some co-occurring strains at lower abundances (Figs. 4, 5; Supplementary Table S3). Where communities differed the most was in *Arsenophonus* strain diversity and abundance (Figs. 3, 5). For example, there were 6 distinct *Arsenophonus* strains found within *A. asclepiadis* communities that ranged between 50 and 227 fold higher relative abundance compared to *A.* *nerii* and *M.* *asclepiadis*. *A.* *asclepiadis* also harbored 5 distinct *Buchnera* strains across the locations sampled. In addition, *Buchnera* strains varied from 92.1% sequence similarity to several strains differing by only 1–2 base pairs (> 99.2% similarity) (Supplementary Table S7). *Arsenophonus* strains were even more similar to each other, varying from 94.7 to 99.6% similarity (Supplementary Table S7).

## Discussion

The prevailing view of host-associated microorganisms and their role in insect-plant coevolution is rapidly changing. Acquisition of beneficial microbes serves not only to expand the ecological niche of the host, but can add novel weaponry in the adaptive battle between plants and the herbivores that colonize them^3,5,7,8^. Furthermore, insects not only acquire microbes from host plants and the surrounding environment, but these microbial communities are intimately linked^9,56,57^. Disentangling the mechanisms driving variation in herbivore microbial communities and the ecological consequences for host plant specialization is therefore of great interest. In the current study, shifts were observed in the core microbiome of aphid species exhibiting varying degrees of host specialization within the milkweed family. Aphids feeding on milkweed share most bacterial taxa when the broader pool of microbes detected across populations is considered (Fig. 1), but each species harbors unique populations that differ in strain diversity and relative abundance of core heritable symbionts, most notably *Arsenophonus*(Figs. 1b, 3, 5). However, broader patterns in bacterial symbiont diversity that scale with diet breadth were not observed (Figs. 3, 4). Instead, our results provide support for the hypothesized effect of overlapping host plant range and common selective pressures (e.g. plant chemical defenses) leading to similarities in microbiomes across milkweed aphid species.

Host plant specificity can influence herbivore associated microbial communities on multiple levels, including causing changes in functional, taxonomic or strain level diversity or by altering the abundance of individual taxa. The microbiomes of generalists and specialist herbivores could also vary simply due to differences in the contribution of heritable versus environmentally acquired microbes, the latter being more variable and transient. However, diet may in fact generally be a poor predictor of insect bacterial community composition^22,58^, with exceptions only in certain groups of herbivores^59^. For example, Lepidopteran larva have variable gut microbiomes that are likely shaped by the host plant they feed on, but general patterns associated with diet breadth are not observed due to the high turnover rate of these microbial communities^14^. In contrast, recent work in Costa Rican rolled-leaf beetles (*Cephaloleia* spp.) shows diet breadth is linked to microbiome diversity and community structure^24^. Previous research also indicates the composition of aphid microbial communities are structured by host plant^11,15,16,20,25,27,32,34^ and heritable bacterial symbionts are involved in expanding diet breadth^26,28^.

Our results show aphids specialized on a single plant family (*Asclepiadaceae*) that vary in diet breadth have taxonomically similar bacterial communities at the species level (i.e. many taxa in common), but differ in strain diversity and relative abundance of key symbionts (e.g. *Arsenophonus*). Horizontal transfer of facultative symbionts via host plants can occur in aphids^60,61^, which could contribute to similarities in symbiont communities (i.e. shared ASVs) across different species that overlap in host range and/or naturally co-occur on plants. Another possible explanation is exposure to similar nutritional and chemical profiles could homogenize microbiomes of herbivore species feeding on the same host plants. The species in this study are specialists of the *Asclepias* (milkweed) family and therefore may have similar core symbionts due to exposure to closely related host plants. Common milkweed (*A.* *syriaca)* is among the most abundant species in the midwestern United States^43^. Although *A.nerii* and *A.* *asclepiadis* can colonize multiple milkweed species, it is likely the Indiana populations in this study are primarily exposed to *A.* *syriaca*, which could further contribute to similarities in microbial communities. Additionally, phylogenetic relatedness can generally result in closely related aphid species harboring more similar microbiota than distantly related species^21^.

Although milkweed aphid microbiomes were overall similar at the species level, bacterial communities may vary across populations and differences in facultative symbiotic partnerships could contribute to additional ecological variation (e.g. ant tending, parasitism rates, predation). Interestingly, variation in symbiont relative abundance and strain diversity contributed most to differences observed across milkweed aphid microbiomes. In particular, *Arsenophonus* was found in higher abundance in *A.* *asclepiadis* compared to the other two species. *Arsenophonus* is a notorious shape-shifting insect symbiont, known best for reproductive manipulation of its host^62,63^. Most aphid facultative symbionts are found in much lower abundances compared to the obligate nutritional symbiont *Buchnera*, suggesting the unusually high abundance observed in *A.* *asclepiadis* could be linked to symbiont complementarity, as has occurred in other aphid species^64^. It is also possible *Arsenophonus* provides a general fitness boost, similar to what has been observed in the soybean aphid^65^. Finally, differences in symbiont populations could shape milkweed aphid-ant mutualisms, possibly via microbial induced changes to honeydew or emission of chemical compounds that mediate partner attraction. Previous work shows insect social partnerships not only uniquely influence each host's symbiotic microbiome^66^, but that volatile organic compounds produced by aphid-associated microbes play a role in attracting ant mutualists^67^. Among milkweed aphids, *A.* *asclepiadis* is consistently tended by ants and benefits from enhanced protection from predators, while *A.* *nerii* is occasionally ant tended and *M.* *asclepiadis* appears to be a loner lacking ant friends^68^. Based on the current study an intriguing question arises; Does *Arsenophonus* mediate milkweed aphid-ant interactions and thus contribute to observed differences in ant attendance? Although *Arsenophonus* does not appear to influence the intensity of ant attendance in cowpea aphids^69^, it is possible this symbiont has evolved a different ecological role in the case of *A.* *asclepiadis*. However, given the 16s rRNA metabarcoding approach used in this study only provides relative abundances, *Arsenophonus* titer levels will need to be confirmed using additional methods such as quantitative PCR in order to take the first step towards addressing potential functions, including nutritional supplementation or aphid-ant interactions. Detection of additional facultative symbionts (e.g. *Serratia*) also warrants further investigation into symbiotic relationships and functional roles in milkweed aphid biology and ecology.

One unexpected result was the occurrence of a single strain of *Buchnera* in all three aphid species (i.e. ASV 1; see Fig. 2) even after stringent filtering for false positives. This is the dominant strain infecting *A.* *nerii* and is found in significantly higher abundance compared to the other two species (Fig. 2, Supplementary Table S6). The current wealth of research on *Buchnera* shows this primary endosymbiont of aphids lives intracellularly, relies purely on vertical transmission, and has exhibited co-cladogenesis with aphid hosts over millions of years. It is unlikely this strain has been transferred across species, but it is also unclear why our dataset shows higher levels of sequencing “cross-talk” between samples than previously observed (e.g.^50^). One possible explanation could be that taxonomic classification using the 16s rRNA gene is unable to provide strain-level resolution for *Buchnera* in some cases (i.e. multiple strains with identical 16s sequences grouped as ASV1) and therefore additional genomic information is needed to distinguish unique strains found across milkweed aphid species.

The current study is limited in that aphids were collected from a single host plant (i.e. common milkweed) and generalist species with a host range outside the milkweed family (e.g. *Myzus persicae*) were not characterized due to low occurrence in the field. We sampled aphids from a single common host plant rather than multiple milkweed species in order to focus on identifying differences in core heritable symbionts (e.g. presence/absence of taxa, large shifts in relative abundance) and reduce variation introduced by environment and host plant differences. Consequently, our sampling design has limited ability to detect changes in environmentally acquired microbes and does not test for changes in microbiome composition induced by feeding on different host plant species. To gain a more complete understanding of milkweed aphid microbial communities, further work is needed that characterizes microbiota from the broad and narrow specialists (*A.* *nerii & M. asclepiadis*) when feeding on multiple host plant species. Imbalance in sampling across aphid species (e.g. fewer *M.* *asclepiadis* samples, Supplementary Table S1) resulting from natural variation in prevalence could also mean that some microbial variation was missed. Finally, while this study profiled only bacterial symbionts, additional microbes present in the broader aphid microbiome (e.g. fungi) may be affected by differences in host plant range. Recent work shows bacterial communities associated with milkweed leaves and roots are unique to species, which ultimately shapes the gut microbiomes of insect herbivores like monarch caterpillars that feed on them^70^. Additional studies are therefore needed to dive deeper into the role host plant species plays in shaping milkweed aphid symbiont community composition and function, especially potential links between plant defensive chemistry and microbiome assembly. In general, further research investigating the generalist-specialist gradient using herbivores that feed across multiple plant species and families is needed to clarify the extent to which diet breadth shapes microbial communities (e.g.^24^).

In summary, we did not find evidence for a gradient in bacterial community diversity associated with variation in diet breadth for milkweed specialized aphid species. Instead, our results suggest overlapping host plant range and shared hosts can result in selection for common microbes and thus microbiomes with high similarity in overall composition when considering the entire pool of microbes detected at the species level. However, milkweed aphids do harbor bacterial populations that vary in strain diversity and relative abundance of *Arsenophonus*, although a handful of other well-known aphid symbionts were also detected in low abundance. These findings suggest that while diet breadth may not be a major driver of divergence in overall taxonomic composition of aphid symbiont communities, factors such as strain level variation and differences in abundance offer alternative routes to generating adaptive potential. Further research is needed to determine the functional or ecological role played by milkweed aphid facultative symbionts and different co-occurring strains.

## Supplementary Information

Supplementary Information 1.

Supplementary Information 2.

## Data Availability

DNA sequencing data generated and analyzed in this study is available on the NCBI Sequence Read Archive (SRA) repository under accession number PRJNA635683.

## Code availability

All code for statistical analyses and figures are available at https://github.itap.purdue.edu/LaramyEndersGroup/Milkweed-Aphid-Microbiome.

## References

[1] Barbosa P, Krischik VA, Jones CG (1991). Microbial mediation of plant-herbivore interactions.

[2] Berenbaum, M. R. Allelochemicals in insect–microbe–plant interactions; agents provocateurs in the coevolutionary arms race. In *Nov. Asp. Insect-Plant Interact.* (eds Barbosa, P. & Letourneau, D. K.) 97–123 (1988).

[3] Mason CJ, Jones AG, Felton GW (2019). Co-option of microbial associates by insects and their impact on plant–folivore interactions. Plant Cell Environ..

[4] Sugio A, Dubreuil G, Giron D, Simon J-C (2015). Plant–insect interactions under bacterial influence: Ecological implications and underlying mechanisms. J. Exp. Bot..

[5] Hansen AK, Moran NA (2014). The impact of microbial symbionts on host plant utilization by herbivorous insects. Mol. Ecol..

[6] Mendes R, Garbeva P, Raaijmakers JM (2013). The rhizosphere microbiome: Significance of plant beneficial, plant pathogenic, and human pathogenic microorganisms. FEMS Microbiol. Rev..

[7] Pineda A, Zheng S-J, van Loon JJ (2010). Helping plants to deal with insects: The role of beneficial soil-borne microbes. Trends Plant Sci..

[8] Hammer TJ, Bowers MD (2015). Gut microbes may facilitate insect herbivory of chemically defended plants. Oecologia.

[9] Liu H, Macdonald CA, Cook J (2019). An ecological loop: Host microbiomes across multitrophic interactions. Trends Ecol. Evol..

[10] Grunseich JM, Thompson MN, Aguirre NM, Helms AM (2020). The role of plant-associated microbes in mediating host-plant selection by insect herbivores. Plants.

[11] Ferrari J, Darby AC, Daniell TJ (2004). Linking the bacterial community in pea aphids with host-plant use and natural enemy resistance. Ecol. Entomol..

[12] McLean AH, Parker BJ, Hrček J (2016). Insect symbionts in food webs. Philos. Trans. R. Soc. B Biol. Sci..

[13] Giron, D., Dedeine, F., Dubreuil, G. *et al.**Influence of microbial symbionts on plant–insect interactions*. In: Advances in botanical research. Elsevier, pp 225–257 (2017).

[14] Jones AG, Mason CJ, Felton GW, Hoover K (2019). Host plant and population source drive diversity of microbial gut communities in two polyphagous insects. Sci. Rep..

[15] Xu T-T, Jiang L-Y, Chen J, Qiao G-X (2020). Host plants influence the symbiont diversity of Eriosomatinae (Hemiptera: Aphididae). Insects.

[16] Qin M, Chen J, Xu S (2021). Microbiota associated with Mollitrichosiphum aphids (Hemiptera: Aphididae: Greenideinae): Diversity, host species specificity and phylosymbiosis. Environ. Microbiol..

[17] Douglas AE (2013). Microbial brokers of insect-plant interactions revisited. J. Chem. Ecol..

[18] Engel P, Moran NA (2013). The gut microbiota of insects–diversity in structure and function. FEMS Microbiol. Rev..

[19] Chung SH, Scully ED, Peiffer M (2017). Host plant species determines symbiotic bacterial community mediating suppression of plant defenses. Sci. Rep..

[20] Holt JR, Styer A, White JA (2020). Differences in microbiota between two multilocus lineages of the sugarcane aphid (Melanaphis sacchari) in the continental United States. Ann. Entomol. Soc. Am..

[21] McLean AH, Godfray HCJ, Ellers J, Henry LM (2019). Host relatedness influences the composition of aphid microbiomes. Environ. Microbiol. Rep..

[22] Jones RT, Sanchez LG, Fierer N (2013). A cross-taxon analysis of insect-associated bacterial diversity. PLoS ONE.

[23] Najar-Rodríguez AJ, McGraw EA, Mensah RK (2009). The microbial flora of Aphis gossypii: Patterns across host plants and geographical space. J. Invertebr. Pathol..

[24] Blankenchip CL, Michels DE, Braker HE, Goffredi SK (2018). Diet breadth and exploitation of exotic plants shift the core microbiome of tropical herbivorous beetles. PeerJ. Prepr..

[25] Gauthier J-P, Outreman Y, Mieuzet L, Simon J-C (2015). Bacterial communities associated with host-adapted populations of pea aphids revealed by deep sequencing of 16S ribosomal DNA. PLoS ONE.

[26] Wagner SM, Martinez AJ, Ruan Y-M (2015). Facultative endosymbionts mediate dietary breadth in a polyphagous herbivore. Funct. Ecol..

[27] Guidolin AS, Cônsoli FL (2017). Symbiont diversity of Aphis (Toxoptera) citricidus (Hemiptera: Aphididae) as influenced by host plants. Microb. Ecol..

[28] Leonardo TE, Muiru GT (2003). Facultative symbionts are associated with host plant specialization in pea aphid populations. Proc. R. Soc. Lond. B Biol. Sci..

[29] Xu S, Jiang L, Qiao G, Chen J (2020). The bacterial flora associated with the polyphagous aphid *Aphis gossypii* Glover (Hemiptera: Aphididae) is strongly affected by host plants. Microb. Ecol..

[30] Ferrari J, West JA, Via S, Godfray HCJ (2012). Population genetic structure and secondary symbionts in host-associated populations of the pea aphid complex. Evolution.

[31] Brady CM, Asplen MK, Desneux N (2014). Worldwide populations of the aphid *Aphis craccivora* are infected with diverse facultative bacterial symbionts. Microb. Ecol..

[32] Henry LM, Maiden MC, Ferrari J, Godfray HCJ (2015). Insect life history and the evolution of bacterial mutualism. Ecol. Lett..

[33] Simon J-C, Carré S, Boutin M (2003). Host–based divergence in populations of the pea aphid: Insights from nuclear markers and the prevalence of facultative symbionts. Proc. R. Soc. Lond. B Biol. Sci..

[34] Brady CM, White JA (2013). Cowpea aphid (*Aphis craccivora*) associated with different host plants has different facultative endosymbionts. Ecol. Entomol..

[35] Blackman RL, Eastop VF (2008). Aphids on the world’s herbaceous plants and shrubs, 2.

[36] Züst T, Agrawal AA (2016). Population growth and sequestration of plant toxins along a gradient of specialization in four aphid species on the common milkweed *Asclepias syriaca*. Funct. Ecol..

[37] Zytynska SE, Weisser WW (2016). The natural occurrence of secondary bacterial symbionts in aphids. Ecol. Entomol..

[38] Harrison JS, Mondor EB (2011). Evidence for an invasive aphid “Superclone”: Extremely low genetic diversity in Oleander aphid (*Aphis nerii*) populations in the Southern United States. PLoS ONE.

[39] Mooney K, Jones P, Agrawal A (2008). Coexisting congeners: Demography, competition, and interactions with cardenolides for two milkweed-feeding aphids. Oikos.

[40] Groeters FR (1989). Geographic and clonal variation in the milkweed-oleander aphid, *Aphis nerii* (Homoptera: Aphididae), for winged morph production, life history, and morphology in relation to host plant permanence. Evol. Ecol..

[41] Dolan, R. W., Moore, M. E. *Indiana Plant Atlas*. [S.M. Landry and K.N. Campbell (original application development), USF Water Institute. University of South Florida]. Butler University Friesner Herbarium, Indianapolis, Indiana (2022).

[42] McMartin, K. A., Malcolm, S. B. *Defense expression in the aphid Myzocallis asclepiadis*. Final Report. Pierce Cedar Creek Institute, Hastings, MI (2008).

[43] Zaya DN, Pearse IS, Spyreas G (2017). Long-term trends in Midwestern Milkweed abundances and their relevance to monarch butterfly declines. Bioscience.

[44] Binetruy F, Dupraz M, Buysse M, Duron O (2019). Surface sterilization methods impact measures of internal microbial diversity in ticks. Parasit. Vectors.

[45] Gohl DM, Vangay P, Garbe J (2016). Systematic improvement of amplicon marker gene methods for increased accuracy in microbiome studies. Nat. Biotechnol..

[46] Caporaso JG, Lauber CL, Walters WA (2011). Global patterns of 16S rRNA diversity at a depth of millions of sequences per sample. Proc. Natl. Acad. Sci..

[47] Bolger AM, Lohse M, Usadel B (2014). Trimmomatic: A flexible trimmer for Illumina sequence data. Bioinformatics.

[48] Martin M (2011). Cutadapt removes adapter sequences from high-throughput sequencing reads. EMBnet J..

[49] Callahan BJ, McMurdie PJ, Rosen MJ (2016). DADA2: High-resolution sample inference from Illumina amplicon data. Nat. Methods.

[50] Jousselin E, Clamens A-L, Galan M (2016). Assessment of a 16S rRNA amplicon Illumina sequencing procedure for studying the microbiome of a symbiont-rich aphid genus. Mol. Ecol. Resour..

[51] McMurdie PJ, Holmes S (2013). phyloseq: an R package for reproducible interactive analysis and graphics of microbiome census data. PLoS ONE.

[52] Dixon P (2003). VEGAN, a package of R functions for community ecology. J. Veg. Sci..

[53] Love MI, Huber W, Anders S (2014). Moderated estimation of fold change and dispersion for RNA-seq data with DESeq2. Genome Biol..

[54] Wright ES (2016). Using DECIPHER v2. 0 to analyze big biological sequence data in R. R J..

[55] Schliep, K., Potts, A. A., Morrison, D. A. & Grimm, G. W. Intertwining phylogenetic trees and networks (No. e2054v1). PeerJ Preprints (2016).

[56] Hannula SE, Zhu F, Heinen R, Bezemer TM (2019). Foliar-feeding insects acquire microbiomes from the soil rather than the host plant. Nat. Commun..

[57] Gomes SI, Kielak AM, Hannula SE (2020). Microbiomes of a specialist caterpillar are consistent across different habitats but also resemble the local soil microbial communities. Anim. Microbiome.

[58] Malacrinò A (2022). Host species identity shapes the diversity and structure of insect microbiota. Mol. Ecol..

[59] Colman DR, Toolson EC, Takacs-Vesbach CD (2012). Do diet and taxonomy influence insect gut bacterial communities?. Mol. Ecol..

[60] Pons I, Renoz F, Noël C, Hance T (2019). Circulation of the cultivable symbiont *Serratia symbiotica* in aphids is mediated by plants. Front. Microbiol..

[61] Li Q, Fan J, Sun J (2018). Plant-mediated horizontal transmission of *Hamiltonella defensa* in the wheat aphid *Sitobion miscanthi*. J. Agric. Food Chem..

[62] Jousselin E, Cø eur d’Acier A, Vanlerberghe-Masutti F, Duron O (2013). Evolution and diversity of A rsenophonus endosymbionts in aphids. Mol. Ecol..

[63] Nováková E, Hypša V, Moran NA (2009). Arsenophonus, an emerging clade of intracellular symbionts with a broad host distribution. BMC Microbiol..

[64] Chong RA, Moran NA (2018). Evolutionary loss and replacement of Buchnera, the obligate endosymbiont of aphids. ISME J..

[65] Wulff JA, White JA (2015). The endosymbiont Arsenophonus provides a general benefit to soybean aphid (Hemiptera: Aphididae) regardless of host plant resistance (Rag). Environ. Entomol..

[66] Ivens AB, Gadau A, Kiers ET, Kronauer DJ (2018). Can social partnerships influence the microbiome? Insights from ant farmers and their trophobiont mutualists. Mol. Ecol..

[67] Fischer CY, Lognay GC, Detrain C (2015). Bacteria may enhance species association in an ant–aphid mutualistic relationship. Chemoecology.

[68] Smith RA, Mooney KA, Agrawal AA (2009). Coexistence of three specialist aphids on common Milkweed, *Asclepias syriaca*. Ecology.

[69] Katayama N, Tsuchida T, Hojo MK, Ohgushi T (2013). aphid genotype determines intensity of ant attendance: Do endosymbionts and honeydew composition matter?. Ann. Entomol. Soc. Am..

[70] Hansen TE, Enders LS (2022). Host Plant species influences the composition of milkweed and Monarch microbiomes. Front. Microbiol..

