# Supplementary material for: Aphid species specializing on milkweed harbor taxonomically similar bacterial communities that differ in richness and relative abundance of core symbionts
Source: Sci Rep. 2022 Dec 7;12:21127. doi: 10.1038/s41598-022-25554-y (PMC9729595; doi:10.1038/s41598-022-25554-y)
Supplement: Supplementary file 1 — Supplementary Information 1. [file 41598_2022_25554_MOESM1_ESM.docx]

**Supplementary Information**

Fig S1: Relative abundances of additional known aphid facultative symbionts in all 3 species that did not meet abundance threshold of > 2 reads across > 10% samples.

Fig S2: Log10(x+1) normalized counts of “other low abundance” category taxa across all three aphid species at Genus level.
